# Pain-induced effects on the pupillary light response under high and low illumination conditions

**DOI:** 10.3389/fneur.2024.1432638

**Published:** 2024-07-09

**Authors:** Michael Kursawe, Heike Ehrlichmann, Walter Weber, Julia Krabbe, Thomas Kraus

**Affiliations:** Institute for Occupational, Social, and Environmental Medicine, Medical Faculty, RWTH Aachen University, Aachen, Germany

**Keywords:** pupillometry, pupillary light reflex (PLR), pain, sympathetic, parasympathetic, locus coeruleus, cold pressor test

## Abstract

**Objective:**

The present study investigated the impact of two different light intensities on the pain-modulated pupillary light response (PLR). Additionally, it aimed to demonstrate parasympathetic and sympathetic influences on PLR parameters in response to pain, as predicted by functional models.

**Method:**

A total of 24 participants were included in a randomized, repeated-measures design. The PLR was measured in response to both dark and bright light stimuli within two test cycles. Pain was induced using the cold pressor test (CPT), which involved immersing participants' feet in ice water. PLR measurements were taken during baseline and ice-water immersion within each test cycle. The assessed PLR parameters included initial diameter (INIT), latency (LAT), amplitude (AMP), and re-dilation time (ReDIL25). Along with these parameters, heart rate (HR) and pain ratings were also computed and analyzed.

**Main results:**

The CPT caused moderate pain in participants, and the resulting PLR parameters were found to be congruent with the expected parasympathetic and sympathetic nervous system activities. Although the luminance of the stimulus did influence PLR parameters, no interaction with pain exposure was found.

**Significance:**

The results showed that different aspects of pain experienced by an individual, as modulated through the sympathetic and parasympathetic nervous systems, are visible in their pupillary reactions to light. Notably, within the range used in the current study, light intensity did not significantly affect the pain-related PLR effects.

## 1 Introduction

The human pupil, controlled by the iris sphincter and iris dilator muscles, adjusts its size to regulate the amount of light reaching the retina, responding to changes in light conditions (1, 2). In parallel, a growing body of research has indicated that pupil fluctuations correlate with cognitive affordances (3, 4), affective processes (5), and attentional demands (6–8). In particular, the pupillary reaction to light, the so-called pupillary light response (PLR), has been recently considered extensively in terms of factors that influence things beyond simple light characteristics (9). Thus, in the clinical context, the PLR has been evaluated as a diagnostic tool in the field of neurodegenerative diseases (10, 11), used as a marker of exposure to toxic substances and sleep deprivation (12), examined in attentional modulation (8, 13), and researched in the context of pain (14–16). These findings reinforce the value of measuring pupillary reactions to gain insights into processes within the autonomic nervous system and higher cortical functions.

Previous research studies have indicated that PLR intensity depends on brightness (2, 17) and length of the applied light stimulus (18). On a functional level, the dilator muscle is innervated by the sympathetic nervous system (SNS) and the constrictor muscle by the parasympathetic nervous system (PNS) (19). Light incidence on the retina has excitatory connections to both the dilation muscle, via the hypothalamus, locus coeruleus (LC), intermedio-lateral column, and the superior cervical ganglion, and the constriction muscle, via the olivary pretectal nucleus, Edinger-Westphal nucleus, and ciliary ganglion (19). The LC, the central noradrenergic hub, has excitatory projections to the dilator muscle via the intermedio-lateral column and superior cervical ganglion. It also exerts inhibitory influences on the constrictor muscle via the Edinger-Westphal nucleus and ciliary ganglion (19). As a consequence, the constriction phase of the PLR is primarily modulated by the PNS; however, within the re-dilation phase, the SNS is active, and the PNS exhibits reduced activity (20).

Beyond light, noxious stimulation of the skin also has excitatory projections into the LC, significantly impacting the PLR (14, 19). A decrease in the latency of the PLR was observed in patients immediately after surgery, which was attributed to an increased parasympathetic activity related to the pain level (21, 22). Furthermore, the area under the curve was used to detect pain, which was measured via a behavioral pain scale (23). In pediatric patients, faster constriction times and an overall increased pupil diameter were shown to be associated with pain levels (24). One widely used method to induce pain within an experimental setting and trigger a sympathetic response is known as the cold pressor test (CPT), where participants are required to immerse their feet or hands in ice water (25, 26). The resulting moderate pain levels led to a moderate increase in the heart rate (27). Similar effects on the heart rate were found in studies using hot water immersions (28) or heat stimuli (29). The cold-induced pain led to a decreased PLR constriction time in female individuals (= faster constriction), whereas in male individuals, the constriction time was prolonged (30). The same pattern was observed for re-dilation time. Therefore, the authors concluded that an increased parasympathetic response in female individuals was responsible for both the decreased constriction and re-dilation times, whereas the prolonged constriction and re-dilation times in male individuals was explained by increased stress-induced sympathetic activity (30). In addition, a reduced PLR amplitude was found during ice water exposure, which was explained by an increased sympathetic drive of the pupil and its partial withdrawal during light stimulus exposure (31). During repeated ice-water immersions, it was found that a decrease in mean pupil diameter (before the application of the light stimulus) was associated with reduced affective pain quality over repetitions (32).

In contrast, PLR re-dilation and pain intensity ratings were constant during repeated immersions (32). To summarize, in terms of pain, slowed pupillary reactivity and reduced PLR amplitudes can be explained by sympathetic enhancement. Generally, fast reactivity and enhanced amplitudes can be associated with increased parasympathetic activity.

The pain stimulus as well as the anticipation of pain elicited by an electric shock showed a reduced PLR amplitude (27). Participants were instructed to either expect an electric shock or not within different blocks, corresponding to “threat” and “safe” blocks, respectively. In threat blocks, an increase in subjective anxiety rating was found to correspond to reduced PLR amplitudes (27). The findings can be applied to anxious patients showing reduced amplitudes but no difference in re-dilation compared to the control group (33). A generalized model was proposed, stating that noxious stimulation itself should lead to a general sympathetic-driven pupil dilation.

In contrast, fear should attenuate the PLR according to parasympathetic inhibition (14). Fear produces projections to the LC via the amygdala that enhance the inhibitory influence on the Edinger-Westphal nucleus and thus reduce the activity of the pupillary constriction muscle via the ciliary ganglion (19). Therefore, a dissociation in pupillary correlates between pain and pain-related fear is suggested (14, 19, 27, 32).

Although it has been demonstrated that characteristics of the PLR are dependent on the nature of the light stimulus itself, especially the stimulus luminance (2, 17, 18), the interaction of light-dependent and pain-induced changes on the PLR is unknown and was not considered in previous research. Moreover, illumination intensity varied across studies. For example, a 2000 Lux light bulb was placed 16 cm before the eyes (32, 34). Others used a light source with an intensity of 0.43 milliwatts per square meter (mW/m^2^) measured at a distance of 1 cm (27). Bakes et al. (33) reported intensities between 0.09 and 180 millicandelas (mcd), whereas stimulus intensities between 10 and 180 μW were reported in studies investigating pain patients (21, 22). A recent study focusing on mental arithmetic and visuospatial tasks pointed out that the PLR amplitude reduction due to increasing workload disappeared when stimulus luminance was increased compared to medium or constant luminance conditions. The authors changed the luminance from a background screen (30.69 Lux) to a medium (32.06 Lux) or strong (41.83 Lux) illumination condition. A decoupling between perceptual processes and the internal attention focus explained the disappearing effect of the amplitude reduction with increasing illumination (35).

The current study aimed to investigate and identify possible interactions between light stimulus luminance and pain-related activity on the PLR. An experiment was designed where two different illumination conditions were combined with a baseline and a pain condition. In addition to sympathetic influence through the elicited pain visible in the PLR re-dilation phase, parasympathetic inhibition through pain-related fear is expected to alter pupil trajectory characteristics.

## 2 Methods

### 2.1 Participants

In this study, 36 participants were initially included. However, measurements could not be completed for two participants due to technical problems. Additionally, the process of measurements was aborted for three participants due to presyncope (1), syncope (1), and excessive blinking (1). Seven participants had to be excluded during data preprocessing because of insufficient illumination. The final sample consisted of 24 participants (10 women) with a mean age of 23.5 years and an average BMI of 23.24 (standard deviation = 2.86). The exclusion criteria were participants with any of the conditions: epilepsy, migraine, neurological diseases, psychiatric diagnoses, and severe eye diseases (glaucoma, retinal detachment); those under medication influencing SNS and PNS or the ability to react and concentrate; those with drug or alcohol abuse habits, those with artificial eye lenses, those with polyneuropathy, those with Raynaud syndrome, and those who are pregnant. All participants underwent a medical anamnesis, and informed consent was obtained. An expense allowance of €40 was paid to the participants. The study was preregistered at the DRKS (00032198) and approved by the Ethics Committee of the Medical Faculty of the RWTH Aachen (EK 23-120). The study also complied with the Declaration of Helsinki.

### 2.2 Study design

**Figure 1:**
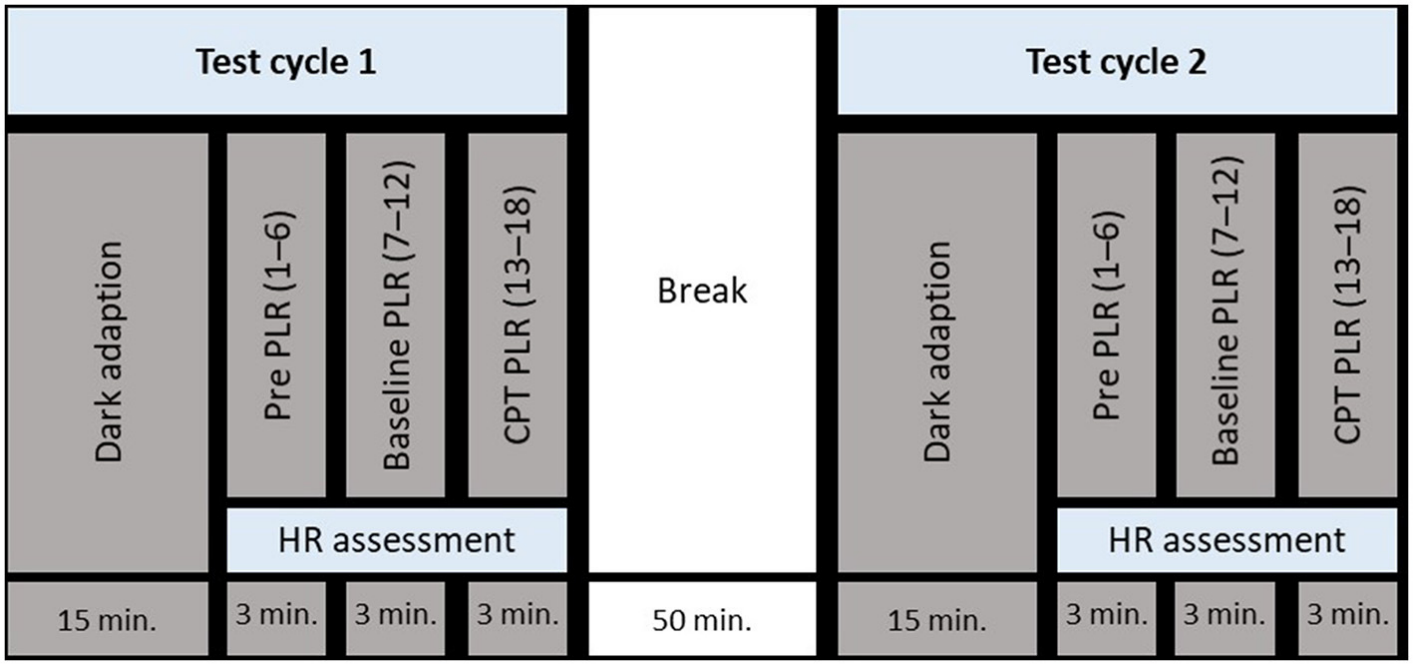
Schematic depiction of the test protocol. For abbreviations, see the Methods section.

The study began with a medical anamnesis and the obtaining of informed consent, after which the experimental setup was explained, and a single PLR measurement was conducted to familiarize the participant with the measurement device. Thereafter, test cycle 1 started with a dark adaptation period followed by pre- (1–6), baseline- (7–12), and CPT PLR measurements (13–18). When PLR measurements were completed, participants were instructed to recover while putting their feet in a warm footbath (around 32 °C) for 5 min. Test cycles 1 and 2 were separated by a 50-min break. An overview of the study design is depicted in Figure 1. Two experimental conditions were randomly assigned to test cycles 1 and 2: (1) dark illumination of the left eye and (2) bright illumination of the left eye. The assignment of the test conditions to test cycles was balanced by gender.

### 2.3 Apparatus and PLR measurement

A high-resolution pupilometer developed and constructed as part of an EU Horizon project (EU-805945) was used in the study, which is equipped with a 1,000 frames per second camera with a resolution of 960 x 900 pixels. With this device, the consensual PLR of the right eye was measured following an illumination of the left eye. A diffuser with a diameter of 50 mm was placed in front of the left eye at a distance of 8 cm. A blue light stimulus was applied for a duration of 30 ms using an LED with a wavelength of 453 nm and a spectral bandwidth of 25 nm. Luminance was set to 1 mcd/m^2^ (*illumination condition*: dark) and 24 mcd/m^2^ (*illumination condition*: bright). The luminance was measured with a Pocket-Lux 2 L luminance meter (LMT GmbH, Berlin, Germany). For calibration and during measurement, the participants' eyes were illuminated with two infrared (IR) light-emitting diodes with a mean wavelength of 940 nm and a spectral bandwidth of 37 nm placed at a distance of 4 cm below each eye.

To take PLR measurements, the participants were instructed to look straight into the mask of the device while resting their heads on a chinrest. Then, the right pupil was focused by the examiner, and a verbal countdown was started. The measurement started at the end of the countdown, and the light stimulus appeared after 100 ms. The overall video recording time was 3,100 ms. The participants were instructed not to blink during the 3,100 ms measurement. Three consecutive measurements were conducted without removing the head from the mask, lasting ~70 s. Thereafter, the participants were instructed to remove their heads and relax for ~30 s. Then, the protocol resumed with the next three measurements. All tests were conducted between 10 am and 1 pm.

### 2.4 CPT, heart rate, and pain rating

For performing the CPT, a mixture of crushed ice and cold water was prepared in a zinc tub directly before the start of the dark adaptation. The temperature of the ice water was ~1.5 °C, and the filling level was 12–15 cm. The participants were instructed to immerse their feet in the ice water directly before the start of the 13th PLR measurement. During the 30-s relaxing break after measurement 15, participants' feet were removed from the ice water for 20–30 s. The last three PLR measurements were conducted under ice water exposure. Following the CPT, the participants were asked to rate their pain level via a numerical rating scale (NRS), which ranged from 1 (no pain) to 10 (severe pain). Values between 4 and 6 refer to a moderate pain level.

During all PLR measurements, continuous heart rate (HR) was recorded using a Polar Vantage V2 device (Polar Electro Oy, Kempele, Finland). The recording was started and stopped by the examiner. Due to technical issues, the HR recording of four participants was not completed.

### 2.5 Data processing and statistical analysis

Four parameters from the pupillary response curve were calculated for further analysis: The initial diameter of the pupil measured in mm (INIT), the latency until the onset of a reaction after the light impulse, measured in ms (LAT), the minimum pupil diameter reached and the amplitude of the reaction, calculated from the minimum pupil diameter reached, measured in mm (AMP), and the time it took for the pupil to re-dilate by 25% of the amplitude after constriction, measured in ms (ReDIL25). INIT is calculated by taking the average pupil diameter during the light impulse (30 ms). LAT is calculated by fitting a linear function to the pupil diameter over a period of 250 ms around the light impulse (starting 50 ms prior to the start of the light impulse and extending 200 ms past it), assuming a roughly normal noise distribution around that trajectory, and identifying the point in time from which the pupil diameter starts to significantly deviate from this assumption.

**Figure 2:**
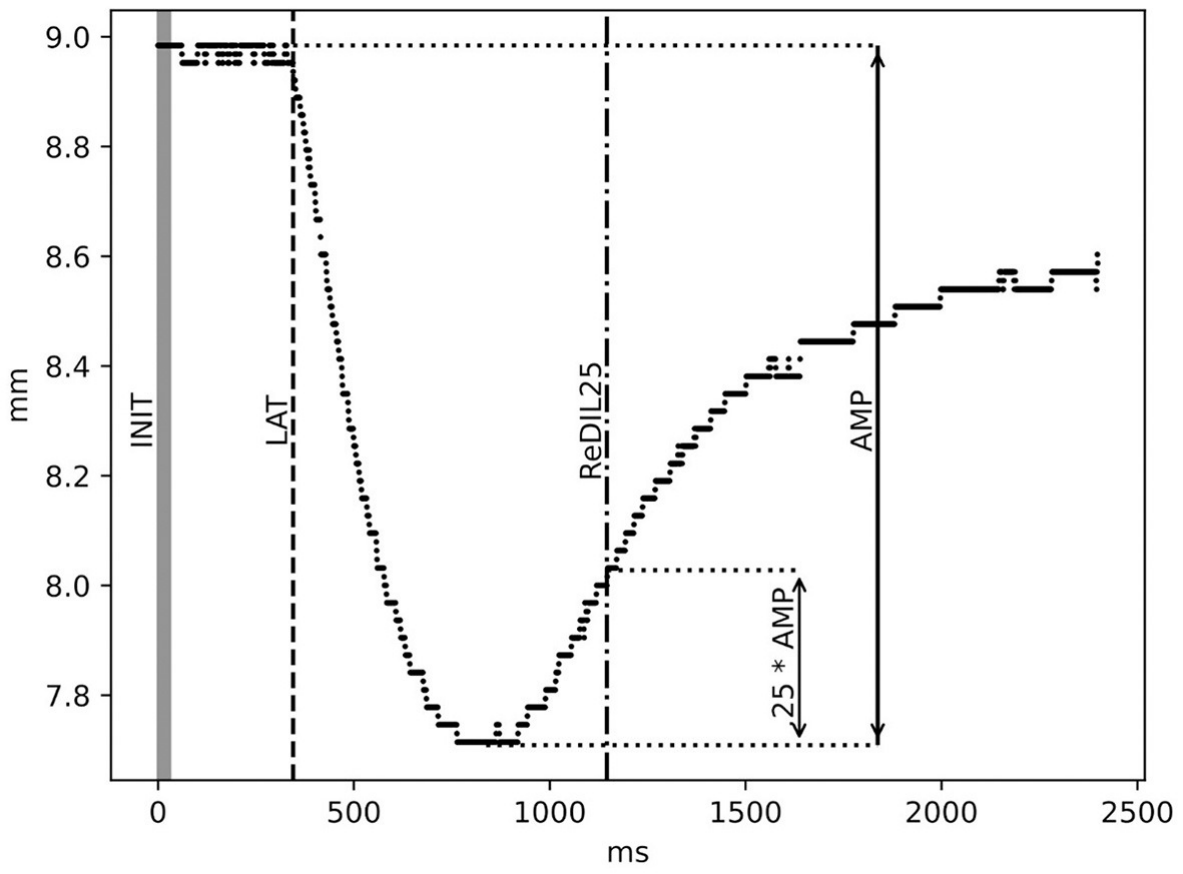
Illustration of PLR parameters: initial diameter in mm (INIT), latency in ms (LAT), amplitude in mm (AMP), and 25% re-dilation in ms (ReDIL25). For a detailed description of the calculation, see the Methods section.

To calculate AMP, the minimum diameter (MIN) was determined by fitting a quadratic function in a region of 400 ms around the minimum in the data, which is typically found in a rough window where the reaction minimum is expected to occur (300 ms to 1000 ms after the light impulse). The minimum is then calculated as the lowest point of this quadratic interpolation. AMP was then calculated as the difference in the pupil diameter between the LAT and the minimum. ReDIL25 refers to the time point at which the average pupil diameter in a 10-ms sliding window crosses MIN + 0.25^*^AMP in the re-dilation period. A graphical illustration of the parameters is shown in Figure 2. The HR parameter was calculated as the average across the period of exposure-related PLR measurements. The period length was ~3 min for each of the six baseline PLR measurements and the six PLR measurements during the CPT.

For statistical analyses, PLR parameters, INIT, LAT, AMP, and ReDIL25, as well as HR measures, were inserted into 2 × 2 repeated measures analyses of variances (rm ANOVAs) with factors *exposure* (baseline and ice water) and *illumination condition* (dark and bright). An α-level of 5% was considered statistically significant. To consider a connection between re-dilation and pain levels, the correlational analysis between ReDIL25 and NRS ratings was conducted using Spearman's correlation coefficient (ρ).

## 3 Results

**Figure 3:**
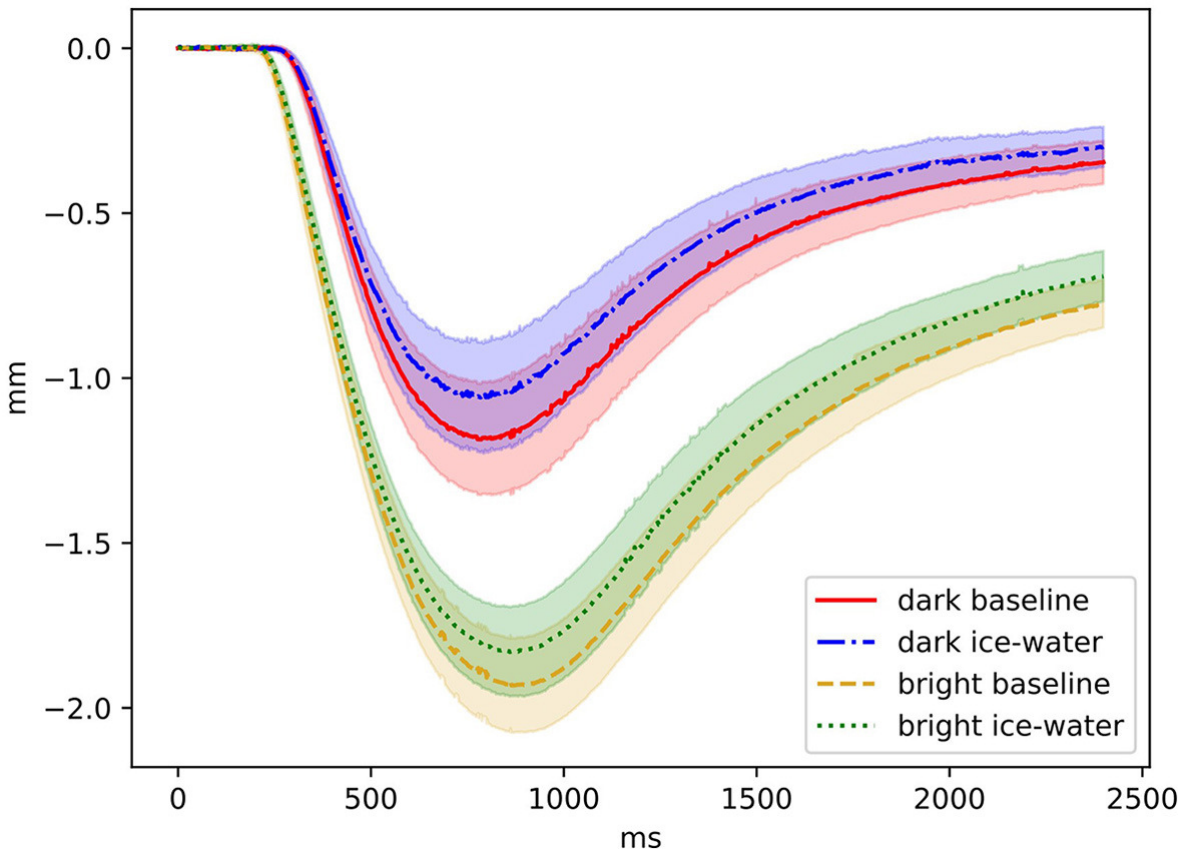
Pupil trajectory levels of factors *illumination condition* and *exposure* are shown. The onset of the illumination was at 0 ms and lasted 30 ms. Relative changes in millimeters (mm) are shown as a function of time in ms. Confidence bands are marked by shaded areas.

The overall mean value of the NRS rating was 5.42 (standard deviation SD = 1.09), which refers to a moderate pain level. Applying a Wilcoxon statistic, between dark (5.5; 1.1) and bright (5.3; 1.09) *illumination condition*, no significant difference was found in the pain levels *(p* > 0.36). Descriptive values of PLR parameters and HR are displayed in Tables 1, 2 shows rm ANOVA results. Significant main effects for *illumination condition* and *exposure* were found in all PLR parameters, whereas the interaction effects of both did not reach significance. For the factor *exposure*, INIT increased during the ice-water exposure and AMP decreased compared to the baseline. LAT was prolonged, and ReDIL25 was significantly shortened during ice-water exposure. Regarding the factor *illumination condition*, INIT and AMP increased as expected during bright illumination. LAT decreased and ReDIL25 increased during bright illumination compared to dark illumination. The influence of the experimental manipulation on pupil trajectories is shown in Figure 3. HR was very similar between the levels of *illumination condition* but increased significantly during ice-water *exposure* compared to baseline. To investigate possible gender and order influences, both factors were included as covariates in all rm ANOVAs, revealing no significant influences (all *p* > 0.12).

**Table 1:** Mean values and standard deviations (in parentheses) for PLR parameters (*n* = 24) and HR (*n* = 20) for both levels of factors *exposure* (baseline and ice water) and *illumination condition* (dark and bright), respectively.

***Exposure***	**Baseline**		**Ice-water**	
***Illumination condition***	**Dark**	**Bright**	**Dark**	**Bright**
**Parameter**				
INIT (mm)	8.04 (0.67)	7.91 (0.71)	8.17 (0.61)	8.06 (0.70)
LAT (ms)	302 (34)	236 (16)	306 (31)	244 (16)
AMP (mm)	1.15 (0.40)	1.90 (0.33)	1.02 (0.40)	1.80 (0.33)
ReDIL25 (ms)	317 (45)	433 (69)	299 (44)	415 (56)
HR (bpm)	74 (11)	75 (11)	87 (13)	87 (15)

For parameter descriptions, see the “Methods” section.

**Table 2:** rm ANOVA results for PLR parameters and HR.

**Parameter**	**n**	***Exposure***		***Illumination condition***		***Exp**. × **Illum***.	
		***p***	η^2^	***p***	η^2^	***p***	η^2^
INIT	24	**< 0.001**	0.54	**0.002**	0.35	0.51	0.019
LAT	24	**0.019**	0.22	**< 0.001**	0.9	0.43	0.027
AMP	24	**< 0.001**	0.62	**< 0.001**	0.94	0.46	0.024
ReDIL25	24	**0.001**	0.37	**< 0.001**	0.86	0.89	0.001
HR	20	**< 0.001**	0.71	0.88	0.001	0.78	0.004

Significance levels (*p*) and effect sizes (η^2^) are displayed along with the sample size (n) for the factors *exposure* (baseline and ice water), *illumination condition* (dark and bright), and the interaction of both factors. Significant results are printed in bold. For parameter descriptions, see the Methods section.

A correlational analysis between all difference values (baseline–ice-water) of both levels of the factor *illumination condition* and pain ratings reached statistical significance (ρ = −0.31, *p* < 0.05). This negative correlation shows that shortened timing in re-dilation is associated with increasing pain ratings.

## 4 Discussion

The present study aimed to evaluate the effects of two different illumination intensities on the impact of ice-water-induced pain on the PLR. Additionally, it was hypothesized that sympathetic influence via pain, as well as parasympathetically mediated pain-related fear, modulate parameters of the PLR. Overall, the ice-water exposure elicited a moderate pain level in participants, with no significant difference observed between the different illumination conditions. Although pain exposure influenced PLR parameters and illumination conditions, no interaction effects from either factor were found.

As argued by Korda et al. (35), the vanishing mental load effect in strong illumination conditions, visible as reduced PLR amplitudes, is a consequence of an attentional allocation to the increasing light stimulus. Despite differences in the experimental setup, PLR amplitudes in medium and strong illumination conditions were comparable to those observed under dark and bright illumination in the present study. Presumably, the attentional attraction of the bright stimulus in the present study was insufficient to modulate the PLR since pain was too prominent. Additionally, in the study by Korda et al. (35), the stimulus length was longer at 500 ms, a factor likely facilitating attentional attraction. Since PLR amplitude increases with light intensity, a brighter stimulus in the ‘bright condition' could have significantly influenced the reduction in pain-related amplitude. A similar effect is achievable using very low-intensity stimuli to elicit the pupillary response. Nevertheless, the present data suggest that both parasympathetic and sympathetic responses resulting from pain can be seen in the PLR, regardless of the stimulus intensity.

Bright illumination stimuli were associated with larger PLR amplitudes, shorter latencies, and prolonged re-dilation timing, whereas the initial pupil diameter was significantly smaller than that of dark illumination stimuli. These results are in line with previous research on the basic characteristics of the PLR (2, 17). The decreased initial pupil diameter during bright illumination conditions will likely reflect a reduced adaptation process to the dark-adapted pupil size between the two measurements. Furthermore, ice-water exposure significantly prolonged PLR latency, reduced amplitudes, and shortened re-dilation times. The initial diameter was increased when in pain compared to the baseline condition, which corresponds to previous research (24, 27, 32), reflecting general sympathetic alertness. Since a dependency between latency and amplitude is known (17), the amplitude reduction fits the model outlined by Szabadi (19) predicting a fear/anxiety-driven inhibitory intake from the LC to the Edinger-Westphal nucleus, resulting in reduced parasympathetic activity. Although participants were not required to rate their anxiety in the present study, they verbally reported feeling slightly afraid of the ice-water immersion before the initial and subsequent exposures. Shorter re-dilation times during ice-water exposure, compared to baseline, indicate an increase in sympathetic influence and a decrease in parasympathetic activity, as also predicted by Szabadi (19). This pain-related re-dilation effect is further supported by a significant negative correlation with results of the Numeric Rating Scale (NRS). However, the gender effect on re-dilation times, as reported by Davis et al. (30), which indicated a more pronounced sympathetic response in men than women, could not be replicated in our study. Possible explanations include an overlay of sympathetic and parasympathetic activities within the early re-dilation phase that could obscure any gender-specific effects due to an imbalance of parasympathetic and sympathetic activities. Both the reduction in amplitude and the shorter re-dilation times during ice-water exposure reflect the pain-modulated activity of the autonomous nervous system, as measured by the pupil's response to light.

## 5 Limitations

Although the participants were instructed to take a warm footbath after both test cycles, it is possible that they did not fully recover from their pain levels during the 50-min break. To address this concern, pain ratings and HR were compared between the first and second test cycles, which showed no differences. Additionally, while the order of the illumination conditions was randomly assigned to test cycles 1 and 2, the sequence of baseline and pain exposure was consistently maintained. This consistency was strategic to prevent the influence of pain from altering baseline PLR measurements. Habituation and stabilization of the PLR were achieved through pre-PLR measurements conducted before starting baseline measurements.

In the current study, the dropout rate was notably high (seven participants) due to the low IR intensity used. A very low IR intensity was deliberately chosen to minimize influences on the pupil and to avoid superimposition, especially with stimuli used under dark illumination conditions. Following 15 min of dark adaptation, the onset of IR for calibration and measurement was intended to provoke only a minimal pupillary reaction, aligning with the study's objective to reduce such effects to the barest minimum.

## 6 Conclusion

By measuring the pupillary response to defined light stimuli, an easy and non-invasive measure can be used to gain insights into autonomic nervous system functioning. As predicted, parasympathetic-driven PLR amplitude modulation is associated with fear, whereas sympathetic influences are visible in pupil diameter and re-dilation behavior due to pain levels. The effects are independent of light stimulus intensity, which can therefore be considered irrelevant within the current setup. Nevertheless, in future research, factors such as stimulus duration, onset and offset characteristics, and the color of the applied light stimulus as possible factors for pain-related PLR interaction effects should be considered.

## Data availability statement

The datasets used and/or analyzed during the current study are available from the corresponding author on reasonable request.

## Ethics statement

The studies involving humans were approved by Ethics Committee at the Medical Faculty of the Rheinisch-Westfälischen Technischen Hochschule Aachen (RWTH Aachen). The studies were conducted in accordance with the local legislation and institutional requirements. The participants provided their written informed consent to participate in this study.

## Author contributions

MK: Conceptualization, Formal analysis, Investigation, Methodology, Writing – original draft, Writing – review & editing. HE: Conceptualization, Data curation, Formal analysis, Investigation, Methodology, Writing – review & editing. WW: Data curation, Methodology, Software, Visualization, Writing – review & editing. JK: Investigation, Writing – review & editing. TK: Conceptualization, Supervision, Writing – review & editing.
